# I-BEAT: Ultrasonic method for online measurement of the energy distribution of a single ion bunch

**DOI:** 10.1038/s41598-019-42920-5

**Published:** 2019-04-30

**Authors:** Daniel Haffa, Rong Yang, Jianhui Bin, Sebastian Lehrack, Florian-Emanuel Brack, Hao Ding, Franz S. Englbrecht, Ying Gao, Johannes Gebhard, Max Gilljohann, Johannes Götzfried, Jens Hartmann, Sebastian Herr, Peter Hilz, Stephan D. Kraft, Christian Kreuzer, Florian Kroll, Florian H. Lindner, Josefine Metzkes-Ng, Tobias M. Ostermayr, Enrico Ridente, Thomas F. Rösch, Gregor Schilling, Hans-Peter Schlenvoigt, Martin Speicher, Derya Taray, Matthias Würl, Karl Zeil, Ulrich Schramm, Stefan Karsch, Katia Parodi, Paul R. Bolton, Walter Assmann, Jörg Schreiber

**Affiliations:** 1 Lehrstuhl für Medizinphysik, Fakultät für Physik, Ludwig-Maximilians-Universität München, 85748 Garching b. München, Germany; 2 Lehrstuhl für Experimentalphysik - Laserphysik, Fakultät für Physik, Ludwig-Maximilians-Universität München, 85748 Garching b. München, Germany; 3 Max-Planck-Institut für Quantenoptik, 85748 Garching b. München, Germany; 4 Accelerator Technology and Applied Physics Division, Lawrence Berkeley National Laboratory, Berkeley, CA 94720 USA; 5 Helmholtz-Zentrum Dresden–Rossendorf (HZDR), Bautzner Landstr. 400, 01328 Dresden, Germany; 6 Technische Universität Dresden, 01062 Dresden, Germany

**Keywords:** Acoustics, Experimental nuclear physics, Plasma-based accelerators, Characterization and analytical techniques

## Abstract

The shape of a wave carries all information about the spatial and temporal structure of its source, given that the medium and its properties are known. Most modern imaging methods seek to utilize this nature of waves originating from Huygens’ principle. We discuss the retrieval of the complete kinetic energy distribution from the acoustic trace that is recorded when a short ion bunch deposits its energy in water. This novel method, which we refer to as Ion-Bunch Energy Acoustic Tracing (I-BEAT), is a refinement of the ionoacoustic approach. With its capability of completely monitoring a single, focused proton bunch with prompt readout and high repetition rate, I-BEAT is a promising approach to meet future requirements of experiments and applications in the field of laser-based ion acceleration. We demonstrate its functionality at two laser-driven ion sources for quantitative online determination of the kinetic energy distribution in the focus of single proton bunches.

## Introduction

Laser-plasma accelerator development has been advancing rapidly in the past few decades, opening a new frontier in accelerator physics. High particle numbers at a broad range of relativistic energies, originating from an exceptionally confined region in space and time, are some of the outstanding features of laser-plasma-based ion accelerators^1–4^. Tremendous efforts and progress regarding increasing intensity, repetition rate and various target refinements bring many applications within reach of today’s capability^5–11^. This impressive progress enhances the need for innovative diagnostics development. The direct measurement of the ion kinetic energy distributions that satisfy emerging online evaluation requirements, such as high repetition rate detection at increased ion energies while being robust and EMP (electromagnetic pulse) resistant, is the primary motivation for this work.

Volumetric detectors such as stacks of radiation-sensitive films^12–14^, CR39^15,16^, copper activation^17,18^ and scintillators^19–21^ allow recording of the energy-dependent angular distribution across the full ion bunch energy spectrum. Other methods to date typically rely on sampling a minor fraction of an ion bunch in magnetic and Thomson parabola spectrometers^22–24^. The endeavor to use the ions for applications requires efforts for collecting and guiding a large portion of the diverging ion bunch^25–29^. Due to the energy selectivity typical of particle optics, the need arises to reliably characterize the particles’ full energy distribution from a single bunch directly at application sites^5^ ideally with direct, prompt single bunch readout.

Here we report on a new method for ion energy measurements, relying on analysis of the acoustic wave, generated when an ion bunch dissipates its energy into a water volume^30,31^. The concept of measuring acoustic signal of ionizing particles in water^32,33^ has recently been advanced to verify the range of mono-energetic ion beams^30,31^ in particular in combination with ultrasonic imaging^34,35^ for future clinical applications^36^. The short bunch duration and therefore intense particle flux of laser-accelerated protons allows for the first time a reconstruction of the depth dose distribution and therefore the complete and more complex energy distribution of a single proton bunch (without any averaging or scanning as required in previous work)^34^.

**Figure 1:**
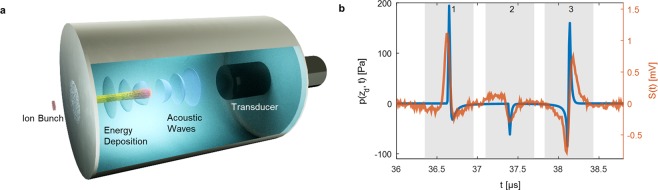
Experimental scheme of I-BEAT. (**a**) The short ion bunch enters the water volume via an 11 µm thick titanium foil (1 cm diameter), depositing its energy in the water and generating an acoustic wave, which is measured via a transducer. This generates a signal as shown in (**b)**, where the orange curve is an example trace for a mono-energetic 9.4 MeV proton bunch measured at the MLL Tandem accelerator and the blue curve represents simulated results, considering an ideal detector with equal conditions.

I-BEAT consists of two parts, the detector that measures the acoustic traces (Fig. 1, details can be found in supplementary material) and the reconstruction algorithm that yields the complete energy distribution. As seen in Fig. 1a the proton bunch enters the cylindrical water chamber from the left (4 cm diameter and length of 10 cm), through an 11 µm thick titanium foil with 1 cm diameter. The ultrasound transducer (Videoscan V311, Olympus), operating in the MHz regime, records the acoustic waves propagating towards the on-axis transducer. If the bunch duration is much shorter than the typical duration of the acoustic wave period (i.e. on the order of µs) the energy deposition can be considered instantaneous. The pressure signal on the axis of propagation at a distance *z* = *z*_*d*_ (position of the ultrasound detector) is then obtained by solving the wave equation (see supplementary material) as follows

1 $$p({z}_{d},t)=\frac{\Gamma {n}_{i}}{4\pi c{\sigma }_{r}^{2}}\frac{\partial }{\partial t}{\int }_{{z}_{d}-ct}^{{z}_{d}+ct}\,{B}_{s}(z^{\prime} ){e}^{-\frac{1}{2{\sigma }_{r}^{2}}[{c}^{2}{t}^{2}-{({z}_{d}-z^{\prime} )}^{2}]}dz^{\prime} ,$$

where ${B}_{s}(z^{\prime} )=\int B({E}_{kin},z\text{'})f({E}_{kin})d{E}_{kin}$ represents the instantaneously generated spread out Bragg curve, *f*(*E*_*kin*_) corresponds to the normalized kinetic energy distribution of *n*_*i*_ ions in a single bunch, *c* is the speed of sound in the medium in which the ions are stopped and Γ is the Grüneisen parameter^37^. For simplicity we assume the transverse profile of the ion bunch in water to be Gaussian with standard deviation *σ*_*r*_.

The blue curve in Fig. 1b exemplarily shows the pressure wave that would be recorded from a mono-energetic 9.4 MeV proton bunch. The signal can be viewed in three segments. In addition to the pulse that propagates directly to the transducer and arrives 36.5 µs after proton impact (segment 1), a second pulse initially propagates in the opposite direction towards the entrance window, and is subsequently reflected back towards the transducer (segment 3). The combination of direct and reflected acoustic signals (segments 1 & 3) images the pressure source from two sides and can thus even be interpreted as a first step towards tomographic analysis. The smaller intermediate signal (segment 2) originates from the energy deposition at the entrance window. Reflections from the side walls of the water housing are temporally well separated and highly attenuated and do not disturb the signal.

Direct comparison and mismatch between the measured (orange) and the ideal pressure signal predicted by Eq. (1) (blue) reveals the need for applying the detector response^38^ correction, i.e. the detector transfer function. Therefore a calibration was performed at the MLL Tandem accelerator in Garching, using well defined proton bunches of 40 ns duration with an energy of 9.4 MeV $(dE/E={10}^{-4})$ (see supplementary material). The calibration allows the determination of the expected observed acoustic trace *S*_*m*_(t) in volts for a given energy distribution $f({E}_{kin})$. The I-BEAT reconstruction algorithm varies $f({E}_{kin}),$ calculates *S*_*m*_ (t) and compares it to the measured curve. With this so called simulated annealing^39^ (see methods), we can iteratively retrieve the discretized energy distribution function $f({E}_{kin})$ and the transverse bunch size *σ*_*r*_ (see supplementary material). A first successful I-BEAT reconstruction of the energy distribution of proton beams with a narrow energy spread has also been shown at the tandem accelerator (see supplementary material).

**Figure 2:**
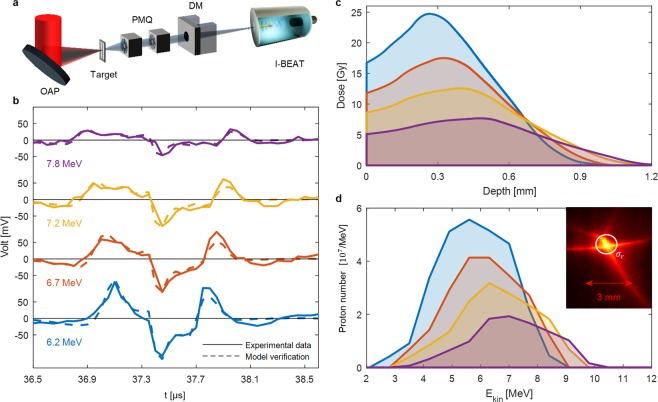
Results for laser-accelerated proton bunches: (**a**) Shows the schematic of the experiment setup. A high power laser (ATLAS-300) is focused with an off-axis parabola (OAP) onto a foil target. Two permanent magnetic quadrupoles (PMQ) are used to focus a short ion bunch. A dipole magnet (DM) is used to remove electrons and low energy ions from the swift ion bunch, which is focused within the ionoacoustic detector. (**b)** Acoustic signals of single proton bunches. The design energies attenuated to 6.2, 6.7, 7.2 and 7.8 MeV on entering the water volume, are set by positioning of the PMQs. The solid line is the measured acoustic signal and the dashed line corresponds to the calculated signal from the retrieved spectrum in (**d)**. (**c**) Depth dose curves corresponding to the different energy settings. The dose on the central axis is $\,D(z)=\frac{1}{\rho }\cdot \frac{1}{2\pi {\sigma }_{r}^{2}}\cdot {B}_{s}(z)$. (**d**) Absolute proton energy distributions of single proton bunches of the different design energy settings in the ion focus. The inset reveals a focal plane image of a single proton bunch at the position of the detector entrance, taken with an image plate. The *σ*_*r*_ marks the lateral extension evaluated with I-BEAT.

After calibration and characterization, I-BEAT was demonstrated using laser-accelerated ion bunches at the Laboratory of Extreme Photonics (LEX Photonics) in Garching near Munich (Fig. 2a)^40^. The ATLAS-300 is a Ti:sapphire laser system delivering 2.2 J energy (on target) within 30 fs at a central wavelength of 800 nm and repetition rate of 1 Hz with 10^20^ *Wcm*^−2^ (on target). By focusing it onto a 250 nm gold foil, a proton bunch with a typically broad TNSA spectrum up to 9 MeV emerged from the surface contamination layers of the plasma source^1,2,41^. A permanent magnetic quadrupole (PMQ) doublet^42,43^ placed closely behind the proton source collected a large portion of this bunch and focused it to the application site outside of the vacuum chamber^5^. The PMQ doublet chromaticity was exploited to focus the design energy to a desired position (application site) by adjusting both the distances to the target and each component of the doublet. The energy distribution of the proton bunch at the focal position was thereby filtered (i.e. narrowed down to a range around the design energy as depicted in Fig. 2d) and not measurable with existing high repetition rate techniques. I-BEAT, placed at application site, enables this measurement. The bunch exited the vacuum chamber through a 50 µm kapton window (1 cm wide in horizontal and 5 cm in vertical dimensions), traversed 3.3 cm of air and the 11 µm thick titanium detector entrance window before reaching the water volume. An additional dipole magnet with an effective field of 150 mT over a length of 0.1 m was employed to ‘clean’ the signal, i.e. directing potential contamination attributed to energetic electrons and low energy ions away from the detector entrance^23^.

Acoustic traces corresponding to single proton bunches with design energies attenuated to 6.2, 6.7, 7.3 and 7.8 MeV at the water tank are presented in Fig. 2b. The spot size at the focus of the laser-accelerated proton bunch was not well defined (a picture taken with an image plate can be seen in the inset of Fig. 2d). While the highest dose is located in an area smaller than *σ*_*r*_ = 1.5 ± 0.2 mm (error is due to the step size of the fitting algorithm) it features also radial caustic shapes at lower dose^44^. Although this overall shape is not Gaussian, the evaluation of the residuals Σ (see methods) for different *σ*_*r*_ produced a comparable result as long as *σ*_*r*_ less than 2.5 mm was chosen. To accelerate the reconstruction process *σ*_*r*_ was fixed to 1.5 mm for the reconstruction of the different energy settings, since this was the best fit for reconstruction of the 6.2 MeV case (see supplementary material). Note that even though the reconstructed diameter fits the extensions of the ion focus well, it is only an estimation relying on the assumption of a symmetric lateral distribution (in our case Gaussian). For more complicated lateral distributions, measurements with more transducers in complementary directions will be required.

Retrieved absolutely calibrated proton energy distributions in the PMQ doublet focal plane are presented in Fig. 2d. The proton number of 10^8^ may seem small but considering that the bunch length in 1.5 m distance is about 15 ns (for a proton bunch with spectrum covering 4 to 8 MeV) and a Gaussian width of 1.5 mm, the proton flux is intense. For the case of 6.2 MeV in Fig. 2, the peak bunch current reaches about 1 mA. As part of the numerical reconstruction, those final retrieved energy distributions (${n}_{i}\,f({E}_{kin})$) are used to calculate an expected signal *S*_*m*_ (t), employing Eq. (1) and the transfer function (dashed curves in Fig. 2b). The excellent conformity of the final retrieved and measured signals demonstrates successful reconstruction of the ion energy distribution. The corresponding on-axis depth dose distribution in Fig. 2c is of particular interest for biomedical application and is a natural byproduct of I-BEAT.

We note that there currently exists no other established method, to which we could reliably compare our results at the presented proton energies (~7 MeV) with similar energy resolution in a focused beam. Therefore we conducted another experiment at the Dresden Laser Acceleration Source (Draco) petawatt laser at Helmholtz-Zentrum Dresden-Rossendorf (HZDR)^45^, enabling a direct comparison of I-BEAT to the well-established radiochromic film (RCF) stack detector and further allowing a demonstration of the feasibility of I-BEAT at a higher fluence level approaching 10^9^ protons per bunch. Particle numbers beyond 10^10^ per bunch are foreseen as realistic (see supplementary material).

**Figure 3:**
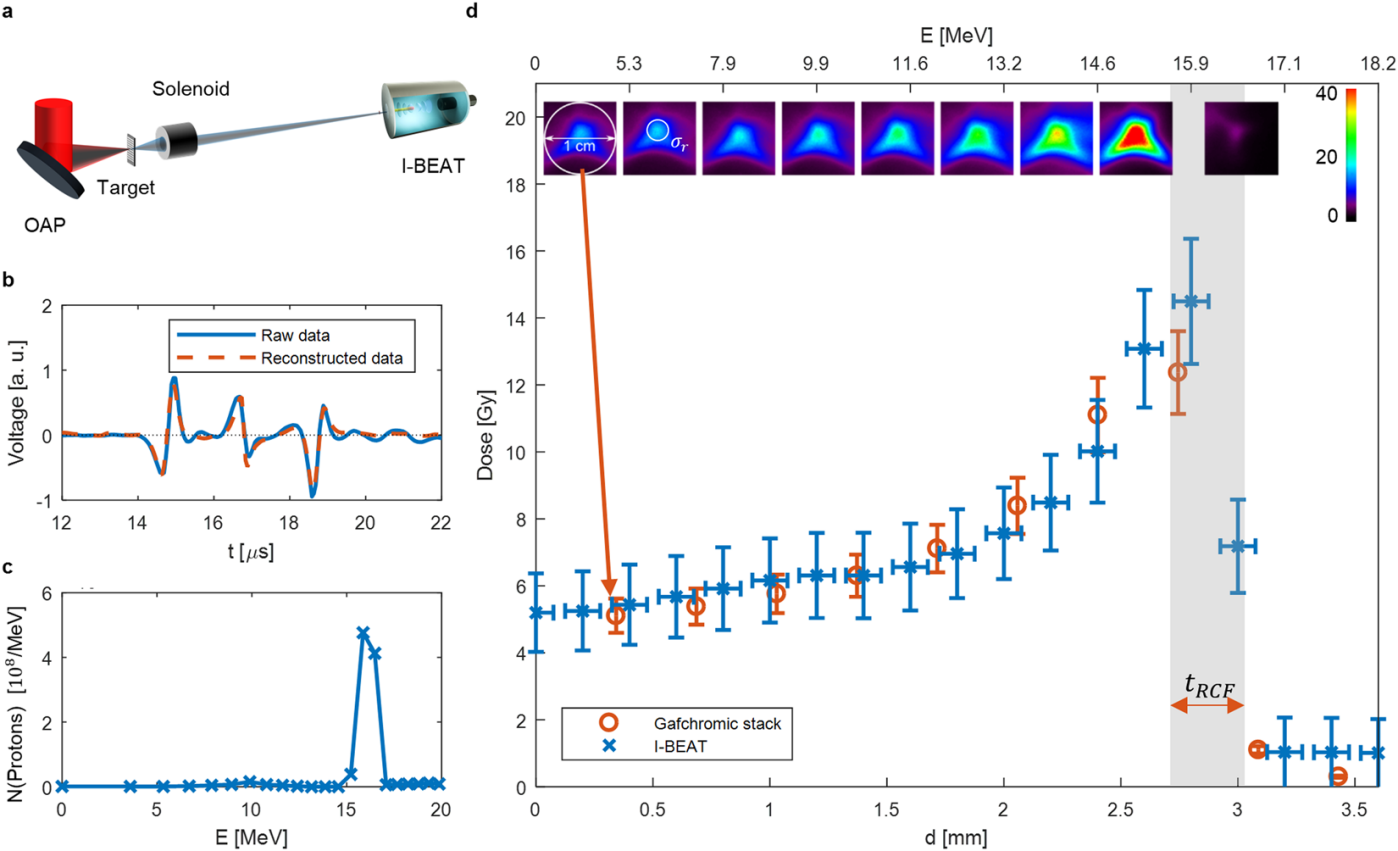
Comparison to RCF stacks at Draco (Dresden): (**a**) is a sketch of the setup. A pulsed solenoid was used to focus a certain design energy. (**b**) is the measured signal and the reconstructed signal according to the energy distribution evaluated in (**c**). (**c**) shows the final reconstructed proton spectrum. (**d**) is the depth dose distribution determined by I-BEAT compared to the one obtained by an RCF stack. The corresponding layers of the stack are depicted and the dose (colour coded in Gy) is evaluated over a circle of 1 cm diameter (entrance of the detector) for both the RCF stack and I-BEAT. The evaluated Gaussian profile determined by I-BEAT yields $\,{\sigma }_{r}=3.0\pm 0.2$ mm and fits very well to the dose of the RCF stack (shown in the second film picture of the stack). The upper axis shows the corresponding proton energy of different penetration depths. The error bar of I-BEAT on the y-axis is due to the calibration of the detector and the noise of measured signal. The error bar of the RCF stack is caused by calibration uncertainties^50^. The grey bar *t*_*RCF*_ illustrates the thickness of one RCF and thus the spatial resolution.

The Draco laser is a Ti:sapphire petawatt laser system, capable of delivering an energy of up to 30 J on target within 30 fs at a repetition rate of 1 Hz. Here it was operated at reduced, 12 J on target, due to a temporal pulse cleaning via a plasma mirror^46^. It was focused onto a 200 nm thin plastic foil, generating a proton bunch with a typically broad TNSA spectrum with energies up to 30 MeV. A pulsed solenoid^29,47^ was used to focus a design energy into the detector outside of the vacuum chamber^6^ (Fig. 3a, see methods). This time, energies up to 30 MeV enabled a comparison with an RCF stack (EBT3 Gafchromic film, calibrated with an X-ray tube). Figure 3 shows a shot for a design energy of about 16 MeV and its comparison to an RCF stack. This energy setting has been chosen to optimize the signal-to-noise ratio recorded by the I-BEAT detector. Figure 3b shows the measured signal and the simulated signal using the evaluated spectrum of Fig. 3c as an input. Figure 3d validates that I-BEAT can reconstruct the depth dose distribution quantitatively. The depth resolution (horizontal spacing between data points) of I-BEAT is due to the sampling rate and transfer function and the error bar due to the limited band width of the transducer (10 MHz). The dose-error of I-BEAT results mainly from the calibration at the Tandem accelerator and noise in the measured signal. For the RCF stack (EBT3-films) an overall accuracy in dose determination better than 5% can be obtained. However, due to higher linear energy transfers (LET) in the Bragg peak region (with respect to the plateau region) of a mono-energetic proton beam, quenching effects might occur, which can lead to higher dose uncertainties. The grey bar *t*_*RCF*_ illustrates the water equivalent thickness and thus the lateral resolution of an RCF. Fitting a 2D Gaussian to the Gafchromic film with the highest dose yields *σ*_h_ of 3.6 mm and *σ*_v_ of 2.2 mm with an average of 2.8 mm. The fitting result of I-BEAT with σ_r_ = 3.0 ± 0.2 mm matches and confirms that the transversal information can be retrieved with a single transducer. Even though the error-bars of the measured dose obtained with I-BEAT are larger than the ones obtained with an RCF stack, the dose matches well. The error-bars of I-BEAT can be reduced with more advance calibration techniques. The energy resolution of I-BEAT at this stage is already better than that of the RCF stack (spatial resolution is nearly doubled, Fig. 3d).

The longitudinal spatial resolution of I-BEAT is in general limited by the frequency of the detector (transducer) and the transfer function. Since the spatial resolution, to first order, is constant along the propagation direction (Fig. 3d), the energy resolution of I-BEAT intrinsically increases for higher ion energies (Fig. 3c). This is in contrast to techniques based on magnetic deflection^22–24^, where the energy resolution decreases with increasing ion energies. For I-BEAT, the energy resolution approaching high kinetic energies is likely limited by energy loss straggling^48^ as for all depth range monitors such as RCF stacks (in general termed position sensitive calorimeters).

We demonstrated I-BEAT for determining the absolute kinetic energy distribution of single focused ultrashort proton bunches using one transducer. In contrast to previous work^34^, no averaging or scanning was required. Even though we demonstrated the use of I-BEAT in air, the detector can also be operated in vacuum without modification (see supplementary material). The harsh conditions typically encountered near laser-plasmas (notably strong electromagnetic pulses)^49^ are key challenges in the design and evaluation of online electronic detectors. In contrast, the relatively low speed of sound results in an inherent µs delay of the acoustic signal, which effectively stores the information. This allows ample time for the decay of prompt undesirable artifacts of the intense laser-plasma interaction, rendering I-BEAT measurements unaffected. While short, intense ion bunches typically saturate detectors (or even cause radiation damage), I-BEAT, using water as a medium, is nearly indestructible and offers a high dynamic range from 10^7^ (Fig. 2) to (at least) 10^11^ (see supplementary material) protons/mm^2^ for intense ion bunches. Further key advantages of I-BEAT include compactness, robustness, simplicity of operation and low cost. With its demonstrated merits, especially in certain situations (e.g. focused beams), we see I-BEAT as a complement and promising addition to the pool of ion diagnostics for research and application at existing and upcoming high repetition rate petawatt laser facilities.

Currently I-BEAT allows the reconstruction of the absolute depth-dose distribution at application sites, in particular downstream of energy selective transport and focusing optics. Furthermore the reconstruction of a broad proton spectrum, typically generated in TNSA-based acceleration schemes has been simulated (see supplementary material). In combination with magnetic energy selection optics (including quadrupoles or solenoids) the detailed diagnosis of more complicated particle bunches consisting of mixed species (for example protons and carbons), can be incorporated in the reconstruction algorithm (see supplementary material for an example). The energy range in which I-BEAT operates can be adapted by proper choice of medium and size to accommodate the complete Bragg curve (starting from several MeV as presented in Fig. 2 up to several 100 of MeV protons)^48^. The clear advantage of I-BEAT is its potential for even higher repetition rate systems. The duration of the I-BEAT signal remains shorter than twice the range of ions divided by the speed of sound, for 100 MeV protons this is 100 µs. The I-BEAT detector could thus be advanced to an operation with repetition rates up to kHz. By employing more transducers in additional directions tomographic reconstruction of the 3D dose distribution seems feasible^50^. This is particularly attractive when the particle energy is completely deposited within a sample, in the simplest case water^51^.

## Methods

### Simulated annealing

The method of simulated annealing relies on a random variation of an initial spectrum *f*_*i*_(*E*_*kin*_) to obtain a modified spectrum *f*_*m*_(*E*_*kin*_), and comparing the two predicted acoustic signals *S*_*i*_(*t*), *S*_*m*_(*t*) from the initial and the modified inputs with the measured signal *S*_0_(*t*) by the least squares method. If the residual Σ*m* is smaller than Σ*i*, the algorithm continues with the modified spectrum as the updated input distribution for the next cycle. Otherwise, with probability $exp(\,-\,({{\rm{\Sigma }}}_{m}-{{\rm{\Sigma }}}_{i})/T)$, the modified spectrum is rejected, while with the probability $1-exp(\,-\,({{\rm{\Sigma }}}_{m}-{{\rm{\Sigma }}}_{i})/T)$ the intial spectrum is taken into the next cycle to avoid being caught in a local minimum. *T* is the annealing schedule temperature and is set to 1. After a sufficient amount (few hundred) of iterations, Σ*i* converges to a global minimum value, the final residual.

### Experimental setup at the draco laser

In the experiment, Draco delivered 30 fs pulses with an energy of about 12 J with enhanced temporal contrast using a re-collimating single plasma mirror on target. Using plastic foil targets with a thickness of about 200 nm, the laser drives a TNSA proton source with cut-off energies in the range of 30 MeV. The tunable solenoid magnet^29,47^ is positioned 80 mm behind the target and is therefore able to collect the high energetic part of the beam without particle loss. It acts as chromatic lens and can be used to generate a focus of a desired mean energy at the irradiation site in air about two meters downstream of the target. The energy bandwidth of the transported proton bunch amounts to about 20% (FWHM) at the focus position. For the presented experiment, a mean proton energy of 15.4 MeV was focused into the I-BEAT detector, corresponding to a solenoid current of 12 kA leading to a magnetic field of ca. 10 T, accordingly. This proton energy has been chosen to optimize the signal-to-noise ratio recorded by the I-BEAT detector.

## Supplementary information

Supplementary Dataset 1

## Data Availability

The data that support the plots within this paper and other findings of this study are available from the corresponding authors upon reasonable request. The datasets generated during and/or analysed during the current study are available from the corresponding author on reasonable request.
