# Supplementary material for: I-BEAT: Ultrasonic method for online measurement of the energy distribution of a single ion bunch
Source: Sci Rep. 2019 Apr 30;9:6714. doi: 10.1038/s41598-019-42920-5 (PMC6491586; doi:10.1038/s41598-019-42920-5)
Supplement: Supplementary file 1 — Supplementary Dataset 1 [file 41598_2019_42920_MOESM1_ESM.pdf]

Supplementary Material for

I-BEAT: Ultrasonic method for online measurement of the energy distribution of a single ion bunch

Daniel Haffa*[1], Rong Yang[§1], Jianhui Bin[1,4], Sebastian Lehrack[1], Florian-Emanuel Brack[5,6], Hao Ding[2,3], Franz S. Englbrecht[1], Ying Gao[1], Johannes Gebhard[1], Max Gilljohann[2,3], Johannes Götzfried[2], Jens Hartmann[1], Sebastian Herr[1], Peter Hilz[1], Stephan D. Kraft[5], Christian Kreuzer[1], Florian Kroll[5,6], Florian H. Lindner[1], Josefine Metzkes-Ng[5], Tobias M. Ostermayr[1,4], Enrico Ridente[1], Thomas F. Rösch[1], Gregor Schilling[2], Hans-Peter Schlenvoigt[5], Martin Speicher[1], Derya Taray[1], Matthias Würl[1], Karl Zeil[5], Ulrich Schramm[5,6], Stefan Karsch[2,3], Katia Parodi[1], Paul R. Bolton[1], Walter Assmann[1] and Jörg Schreiber[1,3]

[1]*Lehrstuhl für Medizinphysik, Fakultät für Physik, Ludwig-Maximilians-Universität München, 85748 Garching b. München, Germany,*

[2]*Lehrstuhl für Experimentalphysik - Laserphysik, Fakultät für Physik, Ludwig-Maximilians-Universität München, 85748 Garching b. München, Germany,*

[3]*Max-Planck-Institut für Quantenoptik, 85748 Garching b. München, Germany.*

[4]*Accelerator Technology and Applied Physics Division, Lawrence Berkeley National Laboratory, Berkeley,CA 94720, USA.*

[5]*Helmholtz-Zentrum Dresden–Rossendorf (HZDR), Bautzner Landstr. 400, 01328 Dresden, Germany*

[6]*Technische Universität Dresden, 01062 Dresden, Germany*

This supplementary materials addresses details of I-BEAT that have not been discussed in the paper. The detector itself is described and pictures are shown. The calculation leading to equation (1) is outlined. The calibration and first experiments of I-BEAT, performed at the Tandem accelerator in Garching near Munich[1], is described and the results are shown and discussed. The dynamic range of I-BEAT and thus its behavior at higher particle numbers is further estimated. The data analysis for the retrieval of the energy spectrum is investigated, especially the influence of different σ_r in the situation of the experiment at LEX Photonics is described. Simulations, where I-BEAT is implemented in typical circumstances are shown.

Email: *Daniel.Haffa@physik.lmu.de, §Rong.Yang@physik.lmu.de

Detector Setup

An ionoacoustic detector relies on the detection of the acoustic signal that is generated due to thermal heating of an ion bunch dissipating its kinetic energy in water. Since operation in vacuum was desired, we chose a KF40 vacuum pipe with 10 cm length as water container. A hole of 1 cm diameter at the front plate is covered with an 11 µm thick titanium foil that is airtight and waterproof and functions as an entrance window for the ion bunch. The transducer was attached to the rear flange and positioned in the water sample. We chose a focusing transducer (focal length of 25.4 mm) to enhance the signal. Signals generated in focal distance will have the best temporal resolution while the resolution drops off out of focus. Supplementary Fig. 1c shows the geometry of the detector used at the Laboratory of Extreme Photonics (LEX Photonics). A picture of the transducer is given in Supplementary Fig. 1d and the used amplifier (60 dB, HVA-10M-60-B, FEMTO Messtechnik GmbH) in Supplementary Fig. 1e. Note that all parts in the electronic chain influence the signal response and have to be included in the calibration. Since the motivation for I-BEAT was its implementation in a laser-plasma ion accelerator a setup-picture is shown in Supplementary Fig. 1f. The picture is taken at LEX Photonics in Garching near Munich. It shows the implementation of I-BEAT inside the vacuum chamber. The detector was modified at the experiment at the Draco laser. The length of the tube and thus the distance of the source of the generated sound signal was shortened and now positioned directly in the focal plane of the transducer. This improves the signal-to-noise ratio and the temporal resolution of the detector. Supplementary Fig. 1h is a picture of the setup at the Draco laser.

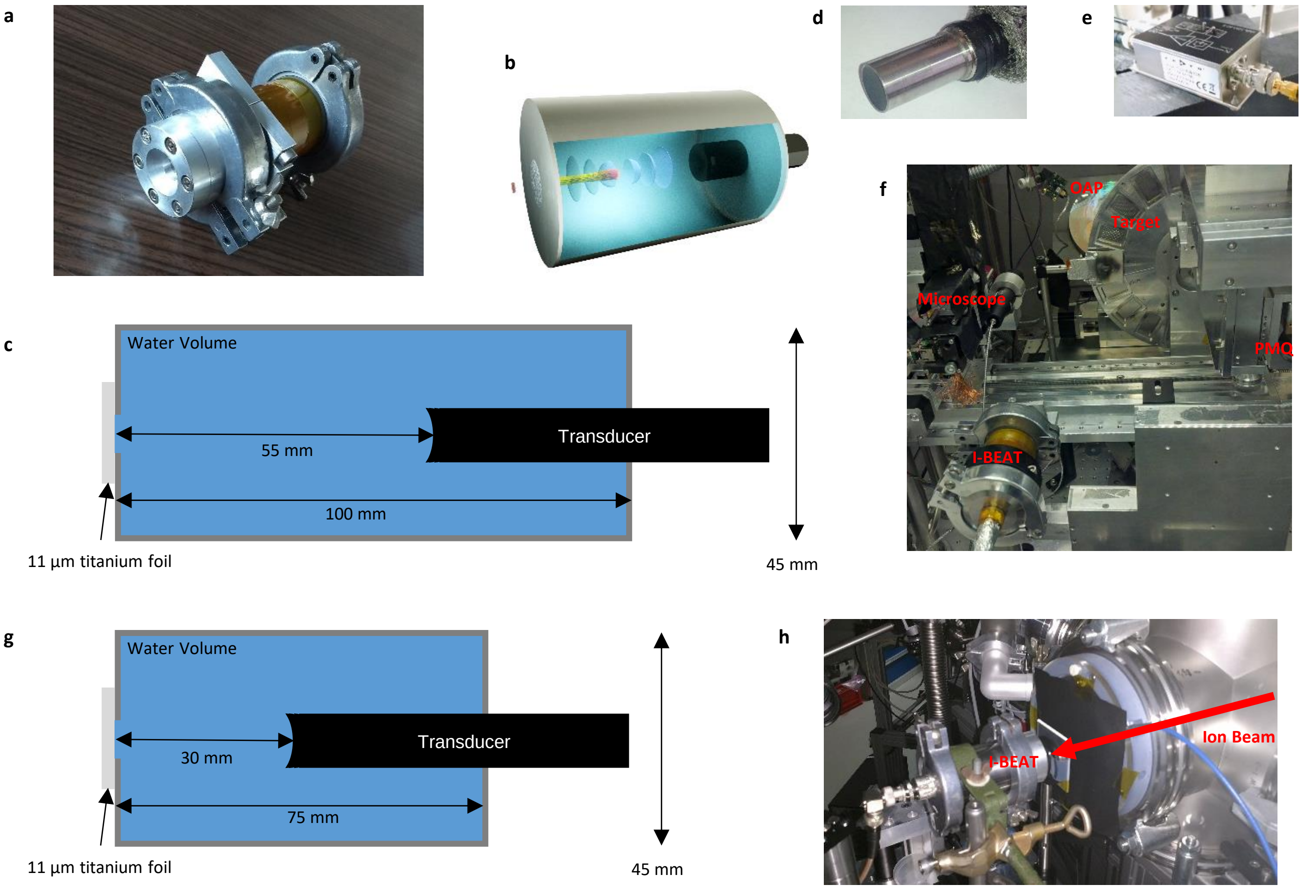

Supplementary figure 1 | Setup of the ionoacoustic detector. a, is a picture of the tube accommodating the water sample (front view). **b,** shows the *detector and depicts the physical process of I-BEAT.* ***c*** *is a sketch emphasizing the dimensions of the used detector at LEX-Photonics.* ***d,*** *is a picture of the transducer that was used during the experiments. It is a focusing transducer with a mean frequency of 10 MHz.* ***e,*** *is the Voltage amplifier used in the experiment.* ***f,*** *shows the setup at the laboratory of extreme photonics in Garching. This shows the implementation of I-BEAT directly in the vacuum chamber. The laser was focused with the off-axis parabola (OAP) onto the target. Two permanent magnet quadrupoles were used to focus the proton bunch into the water volume.* ***g,*** *shows the modified detector that was used at the Draco laser. The detector was shortened in order to accommodate the Bragg peak in the focus of the transducer.* ***h,*** *is a picture of the setup at the Draco laser.*

Calculation

This part describes a more detailed derivation of equation (1)[2,3]. Also the derivation of the reflection coefficient is explained in detail.

By solving the wave equation

$$\left(\nabla^2 - \frac{1}{c^2}\frac{\partial^2}{\partial t^2}\right)p(\vec{r},t) = \frac{-\Gamma}{c^2}\frac{\partial}{\partial t}H(\vec{r},t) \tag{1}$$

with the Grüneisen-parameter Γ in Pa/(J/m³) and the phase velocity of the acoustic wave c, we can approximate the heating function $H(\vec{r}',t') = H_s(\vec{r}')\delta(t')$. This separation is valid since the ion energy deposition can be considered as instantaneous. Denoting the Bragg curve produced by a single ion with a specific initial kinetic energy E_{kin} with $B(E_{kin},z')$ (in *J/m*) and considering a transverse Gaussian distribution with cylindrical symmetry and standard deviation σ_r, the solution on axis at the detector position $z = z_d$ becomes

$$p(z_d,t) = \frac{\Gamma n_i}{4\pi c\sigma_r^2}\frac{\partial}{\partial t}\int_{z_d-ct}^{z_d+ct} B_s(z')\, e^{-\frac{1}{2\sigma_r^2}\left[c^2t^2-(z_d-z')^2\right]}dz', \tag{2}$$

where $B_s(z') = \int B(E_{kin},z')f(E_{kin})\,dE_{kin}$ represents the instantaneously generated spread out Bragg curve and $f(E_{kin})$ corresponds to the normalized kinetic energy distribution of the number of (n_i) ions in a single bunch. The reflectivity of the sound wave at the entrance foil is defined by $R = (Z_M - Z_W)/(Z_M + Z_W)$ with

$$Z_M = Z_0\frac{Z_{load} - iZ_0 tan(k_0d_0)}{Z_0 - iZ_{load}tan(k_0d_0)}, \tag{3}$$

where $k_0 = 2\pi f/c_0$ with c_0 and $Z_0 = \rho_0 c_0$ the speed of sound and sound impedance of the mirror material, and ρ_0 its mass density. $Z_{load} \approx 0$ (vacuum or air), so that $Z_M \approx -iZ_0 tan(k_0d_0)$ and $R = -e^{i\phi}$ with

$$\phi = atan\left(\frac{2Z_wZ_0tan(k_0d_0)}{Z_W^2 - Z_0^2tan^2(k_0d_0)}\right). \tag{4}$$

In the setting relevant for our case $f < 10\ MHz$ and $d_0 = 11\mu m$, $k_0d_0 < 0.13$. The phase term of the reflectivity can thus be neglected and the reflection $R = -1$ corresponds to that of a fixed end such that the polarity of the reflected wave packet is inverted.

Calibration

I-BEAT relies on measuring the acoustic traces originating from pressure changes induced by ions in water. The so called transfer function $T(f)$ connects the pressure waves $p(t)$ with the measured acoustic signals $S_m(t)$ by

$$T(f) = \frac{FT[S_m(t)]_f}{FT[p(t)]_f} \quad (5)$$

and fully depends on the employed transducer and configuration. Thus the transfer function for our detector in units *V/Pa* for our detector had to be calibrated first (Supplementary Fig. 2b)[4].

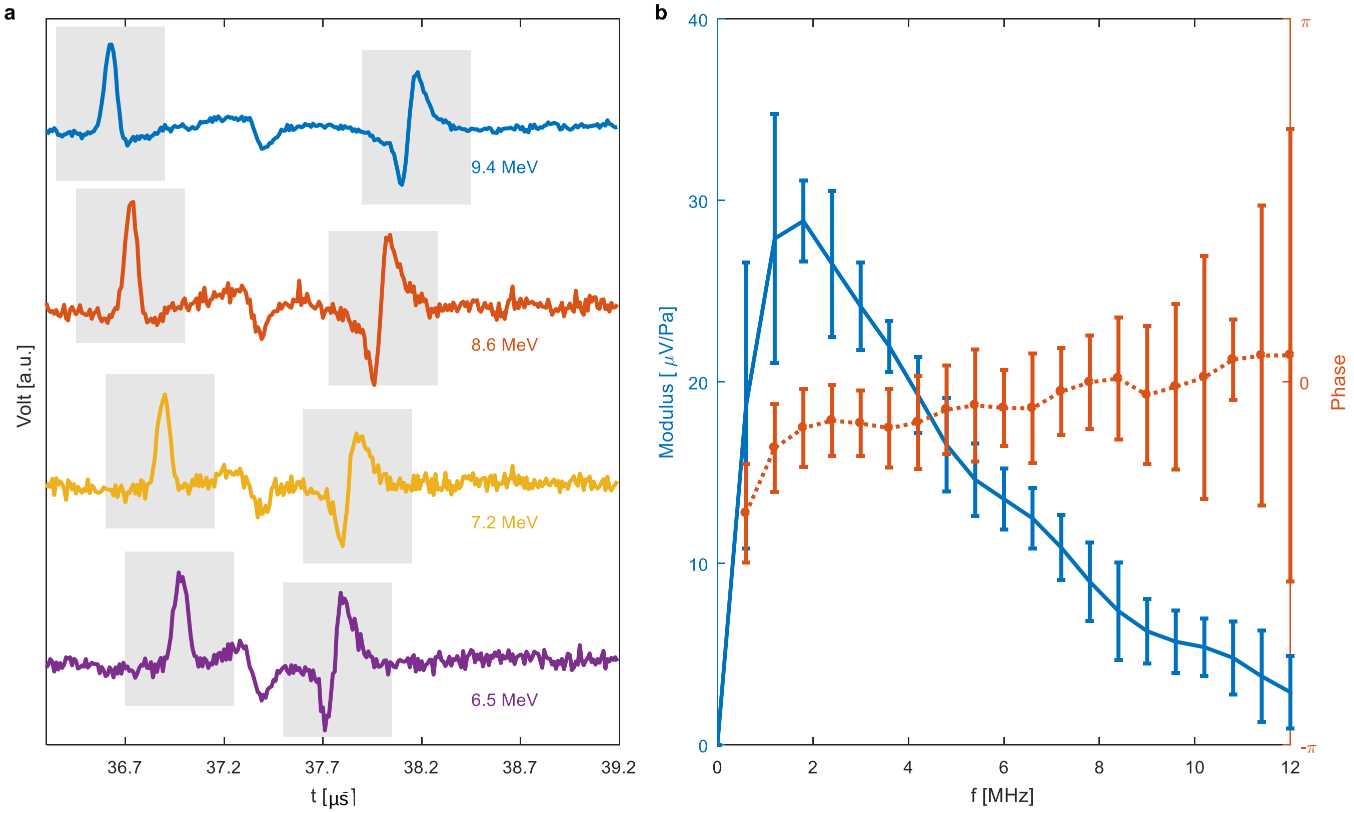

Supplementary figure 2 | Evaluation of the transfer function. a, shows acoustic traces recorded with proton bunches at the Tandem accelerator and outlined regions used for the calculation of the transfer function $T(f)$. **b,** amplitude of the averaged complex transfer function T(f)/N_i in units V/Pa and its phase , where error bars represent maximum deviation of the respective eight independently determined trace segments highlighted in **a**.

The calibration was done using measurements performed at the Tandem accelerator. By inserting aluminum foils of 3 different thicknesses (90, 210 and 270 µm), the central energy entering the detector

was gradually reduced from 9.4 MeV to 8.6, 7.2, 6.5 MeV. Since both the direct and the reflected signal contain the full information, 8 trace segments can be used for determination of the transfer function. Supplementary Fig. 2b shows the averaged transfer function whereas the error bar reflects the maximum deviation of the eight trace segments used for evaluation. The amplitude is quite stable with small error bars. Therefor the same calibration can be used for energies in the range between 7 and 10 MeV. Increasing error bars beyond 10 MHz are due to the use of a 10 MHz transducer. The fact that the frequency peaks around 2 MHz while a 10 MHz transducer is employed can be explained with the effect of geometry spatial response[5]. The used transducer (Supplementary Fig. 1d) has a focal length of 25.4 mm. In our case we measured ultrasound signals with a source more than 50 mm away from the transducer. This out-of-focus operation leads to a degraded temporal resolution consistent with the measured transfer function of Supplementary Fig. 2b. An optimization of the detector response (transfer function) can further improve the resolution and I-BEAT can be adapted to the requirements.

For a quantitative calibration the measurements were also used to estimate the number of protons per proton bunch via $N_i = \int f(E_{kin}) dE_{kin} = I/(e f_{rep})$, where I is the average current that was delivered from the Tandem to the water volume, and $f_{rep} = 5$ kHz is the bunch repetition rate. We estimated, considering the pinhole size of the detector entrance and the spot size of the beam, that 60% of the bunch enters the detector such that current was estimated $= 0.6 \times 7\ nA = 5.2\ nA$. $S_m(t)$ is thus quantitatively connected to the ideal pressure trace $p(t)$ for an arbitrary ion energy distribution $f(E_{kin})$ (predicted by equation (1)) via

$$S_m(t) = IFT[FT[p(t)]T(f)], \qquad (6)$$

First Tests at the Tandem accelerator

Before applying I-BEAT to a laser-plasma accelerator, calibrations and first tests were performed at the MLL Tandem accelerator at Garching, using well defined proton bunches of 40 ns duration with 10 MeV ($dE/E = 10^{-4}$). For a better characterisation of I-BEAT we varied the proton energy. The initial energy of 9.4 MeV at the detector entrance was attenuated by inserting different thicknesses of aluminum in the beam path. The acoustic signals deriving from different energies were measured and are shown in Supplementary Fig. 3a, where each trace represents an average of 100 proton bunches.

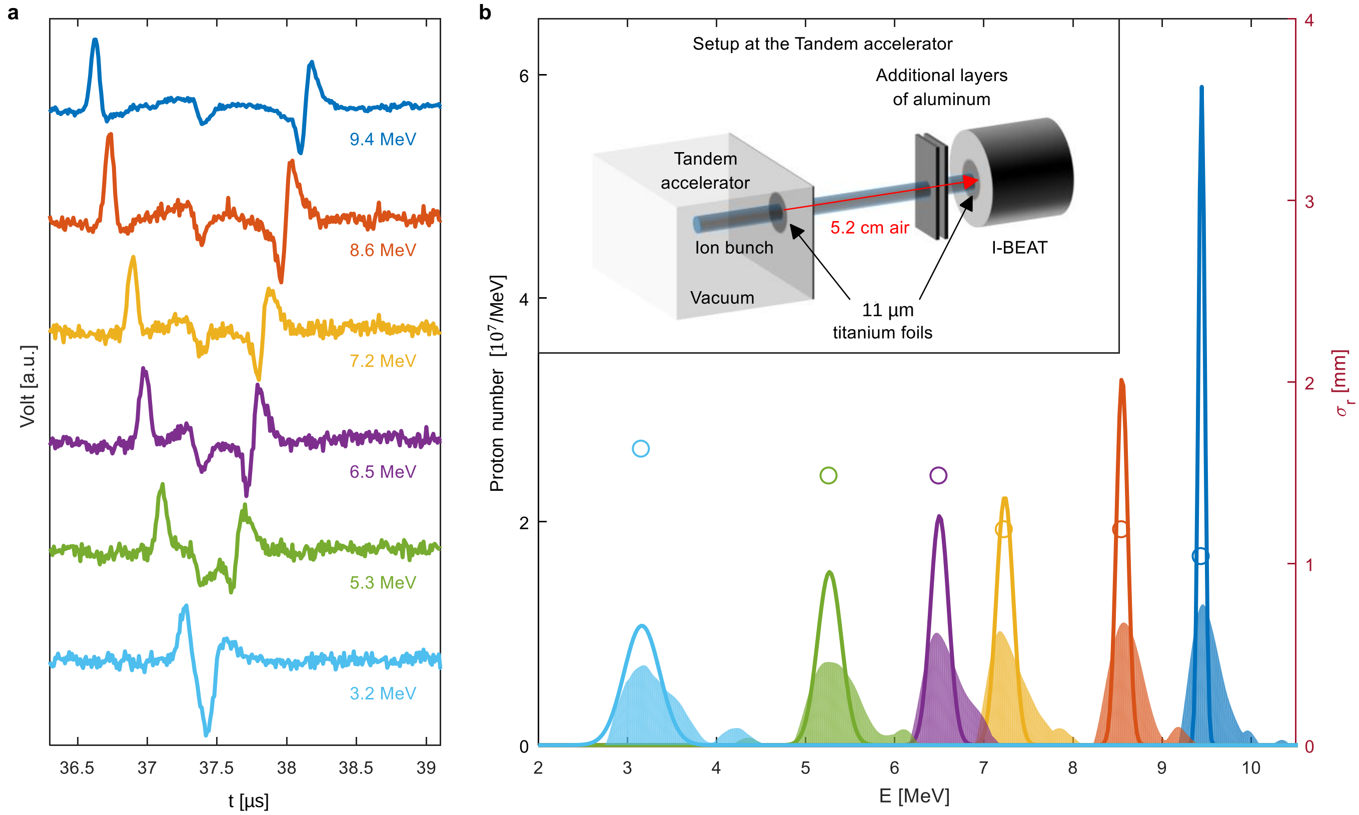

Supplementary figure 3 | Results for Tandem-accelerated proton bunches. a, Acoustic traces of 6 different proton bunch energies at the detector entrance recorded at the Tandem accelerator. Each trace represents an average of 100 proton bunches. **b,** Comparison of proton energy distributions simulated (curves) and retrieved data (filled), as well as reconstructed beam with standard deviation σ_r (circles), of the measured acoustic traces in **a**. The inset of **b** sketches the setup at the Tandem accelerator.

Supplementary Fig. 3b compares the retrieved results to the energy distributions calculated via SRIM (2013)[6] by taking into account the absorber material in the beam path The theoretically expected kinetic energy distributions of protons at the position of the I-BEAT detector were calculated by SRIM, employing a mono-energetic beam with 10 MeV, passing through 11 µm titanium (the exit window of the accelerator vacuum), 5.2 cm air, and 11 µm titanium (the entrance of the water tank). While the maxima of the energy

distributions (simulated and measured) agree within 1%, the larger energy spread and accordingly lower peak value of the experimental data can be explained in terms of the transfer function and its influence onto the resolution.

The transfer function expresses the limit of temporal response and thus linearly affects the longitudinal spatial resolution. Since the spatial resolution, to first order, is constant along propagation direction, the energy resolution intrinsically increases for higher ion energies and is only limited by energy loss straggling for high kinetic energies. We consider the effect of the transfer function on the FWHM of a mono-energetic ion peak to set the resolution limit. Our transfer function yields resolutions of 1.0 MeV at 5 MeV and 0.6 MeV at 10 MeV[4] (increasing resolution towards higher energies). These resolution limits of I-BEAT when applied to narrow energy spread proton bunches at low energies (< 20 MeV) are visible in Supplementary Fig. 3b. The retrieved transverse beam size σ_r increases as expected with increasing aluminum thickness (i.e. decreasing bunch energy) due to the associated transverse straggling that becomes increasingly prominent.

Estimation on the behavior of the detector at higher fluences

The detector is capable of measuring really high particle fluxes. Since this scaling could not be measured so far, the expected temperature increase is estimated in this section, starting with a derivation of Boyle's law:

$$\frac{dV}{V} = -\kappa\delta p + \beta\delta T, \qquad (7)$$

with kappa being the isothermal compressibility and beta the volume expansion coefficient. δp and δT are the changes in pressure and temperature respectively. As we consider only adiabatic heating in ionoacoustics, the volume expansion is neglected and only the transfer from temperature gradient to dynamic pressure is considered. With the use of the specific heat capacity, the following expression can be derived:

$$\delta p = \frac{\beta}{\kappa C_v m}\delta E, \qquad (8)$$

where C_v is the isochoric specific heat capacity, m the mass of the heated area and δE the applied energy as heat. This conversion of energy to pressure is material depended and is quantified with the dimensionless Grüneisen parameter Γ:

$$\Gamma = V\left(\frac{\partial p}{\partial E}\right) = \frac{\beta}{\kappa\rho C_v} = \frac{\beta c^2}{C_p}, \qquad (9)$$

where ρ is the material density, c the speed of sound and C_p the isobaric specific heat capacity. In order to estimate, weather a very high particle number will significantly change the linearity of the energy transfer to dynamic pressure, the expected temperature increase and the change in the Grüneisen parameter is investigated. For liquid water and the temperature T in degrees Celsius, the Grüneisen parameter is well approximated by:

$$\Gamma_w(T) = 0.0043 + 0.0053T. \qquad (10)$$

In independent measurements at the MLL Tandem accelerator, the pressure from 20 MeV protons and $3\text{x}10^6$ $protons\ /mm^2$ was measured with a calibrated, broadband needle hydrophone (Precision Acoustics, UK). Measured at different distances from the source, the pressure at source level was extrapolated to 115 Pa, which in this case corresponds to a temperature gradient of 0.14 mK. Assuming a linear dependence, a temperature gradient of 4.2 K can be expected for 10^{11} protons/mm^2 per bunch.

Based on the approximation for the Grüneisen parameter given above, we derive the following values:

$$\Gamma_w(25) = 0.1368, \Gamma_w(29) = 0.158, \Delta\Gamma_w = 0.0212\ , \qquad (11)$$

which corresponds to a relative change of 15%, in absolute particle numbers, over several orders of magnitude. We can thus say that the detector will also work at much higher particle numbers.

Data Analysis

This section describes the algorithm of simulated annealing[7] in a bit more detail and discusses the influence of different beam diameters onto the retrieval in the case of the laser-accelerated ions.

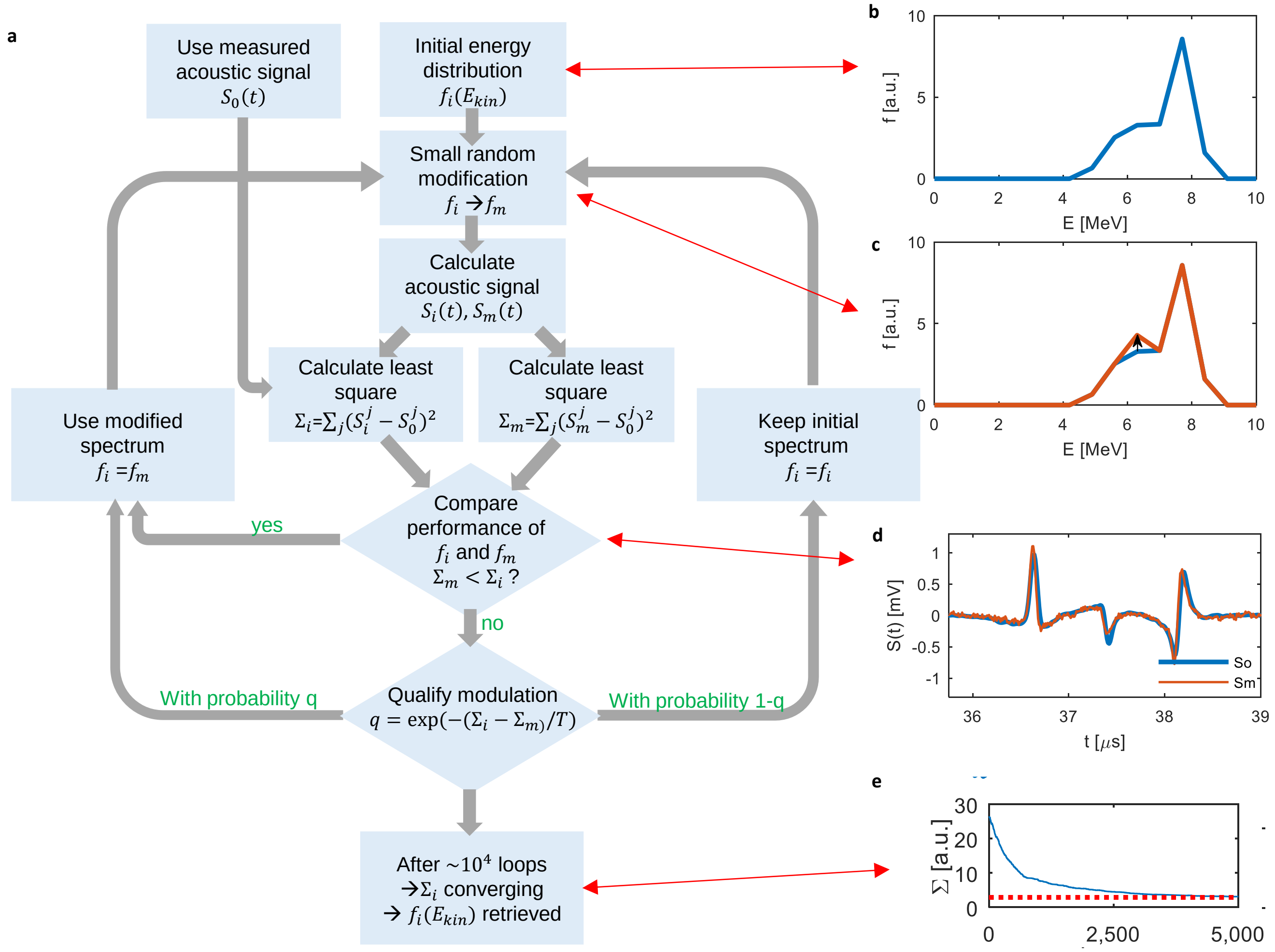

Supplementary figure 4 | Workflow of simulated annealing. **a,** The workflow of simulated annealing is shown. **b,** The initial spectrum f_i can be guessed or started with a flat distribution. **c,** Small modulation to the initial spectrum is done (f_m). **d,** Acoustic signals are calculated and the performance is compared. **e,** $\Sigma_i$converges after about 10^4 loops.

The method of simulated annealing relies on varying an initial spectrum $f_i(E_{kin})$ (a little change applies to the estimated spectrum, and both its position and amplitude are decided by pseudo-random generators) to obtain a modified spectrum $f_m(E_{kin})$. As a starting point $f_i(E_{kin})$ was chosen to be zero for all energies. Typically, the maximum of the amplitude modification is set to be smaller than 1 % of the maximum of f_i. With the input of the initial and the modified spectrum in eq. 1 the predicted acoustic signals $S_i(t), S_m(t)$ are calculated and compared to the acoustic signal $S_0(t)$. The residuals

$\sum_i = \sum_j (S_i^j - S_o^j)^2$ and $\sum_m = \sum_j (S_m^j - S_o^j)^2$ are calculated employing the least squared method. If Σ_m is smaller than Σ_i, the algorithm continues with the modified spectrum as the updated input distribution for the next cycle. For Σ_i smaller than Σ_m , with probability $q = exp(-(\Sigma_m - \Sigma_i)/T)$, the algorithm continues with the modified spectrum, while, with the probability $1 - q$, it is rejected and the initial spectrum is taken into the next cycle. This additional random choice prevents from being caught in a local minimum. T is the annealing schedule temperature and was set to 1. After a sufficient amount of iterations ($\sim 10^4$) the temperature during the iteration would become stable around the temperature global minimum (Σ_i converges), shown in the insets of Supplementary Fig. 5b, Fig. 5c and Fig. 5d , and the obtained proton energy spectrum is the retrieved spectrum. As explained before the ion bunch standard deviation σ_r can be treated as unknown in the retrieval process. In this case the complete process of simulated annealing depicted in Supplementary Fig. 4 is repeated by choosing another σ_r. As a result the final residuals $\Sigma_i(\sigma_r^j)$ after a sufficient number of steps (when a minimum for Σ_i) is found) shows a broad but distinct minimum for a certain bunch diameter (Fig 5a).

Fig. 2 in the main paper shows the reconstruction of laser-accelerated ion data. In this case we fixed the Gaussian bunch standard deviation σ_r to 1.5 mm. Supplementary Fig. 5a shows the residual Σ_i in dependency of the bunch diameter for the case of the design energy set to 7 MeV. We can see that the algorithm converges for σ_r < 2.5mm. The chosen standard deviation for the ion beam diameter of 1.5 mm can thus be explained by the look of the focus image but also the retrieval process. Fig5 b, c and d show the final result obtained for different σ_r. Also the converging residual in dependency to the number of iterations is shown as an inset. At this point the algorithm takes about 10 minutes to retrieve the complete energy spectrum of a certain σ_r. Typically, the retrieval is performed for several σ_r values to find the respective optimum (depending on the knowledge of the bunch distribution). Possible improvements can be obtained by an advanced guess of the initial spectrum $\mathrm{f_i(E_{kin})}$ by taking, for example the central energy of the ion bunch that is contained in the peaks of the acoustic trace, into account. Another possibility would be to use the previous result or model calculations to predict the initial spectrum. Also, further optimizing the retrieval algorithm can decrease the computing time and enable at least a preview of the results shortly after the measurement.

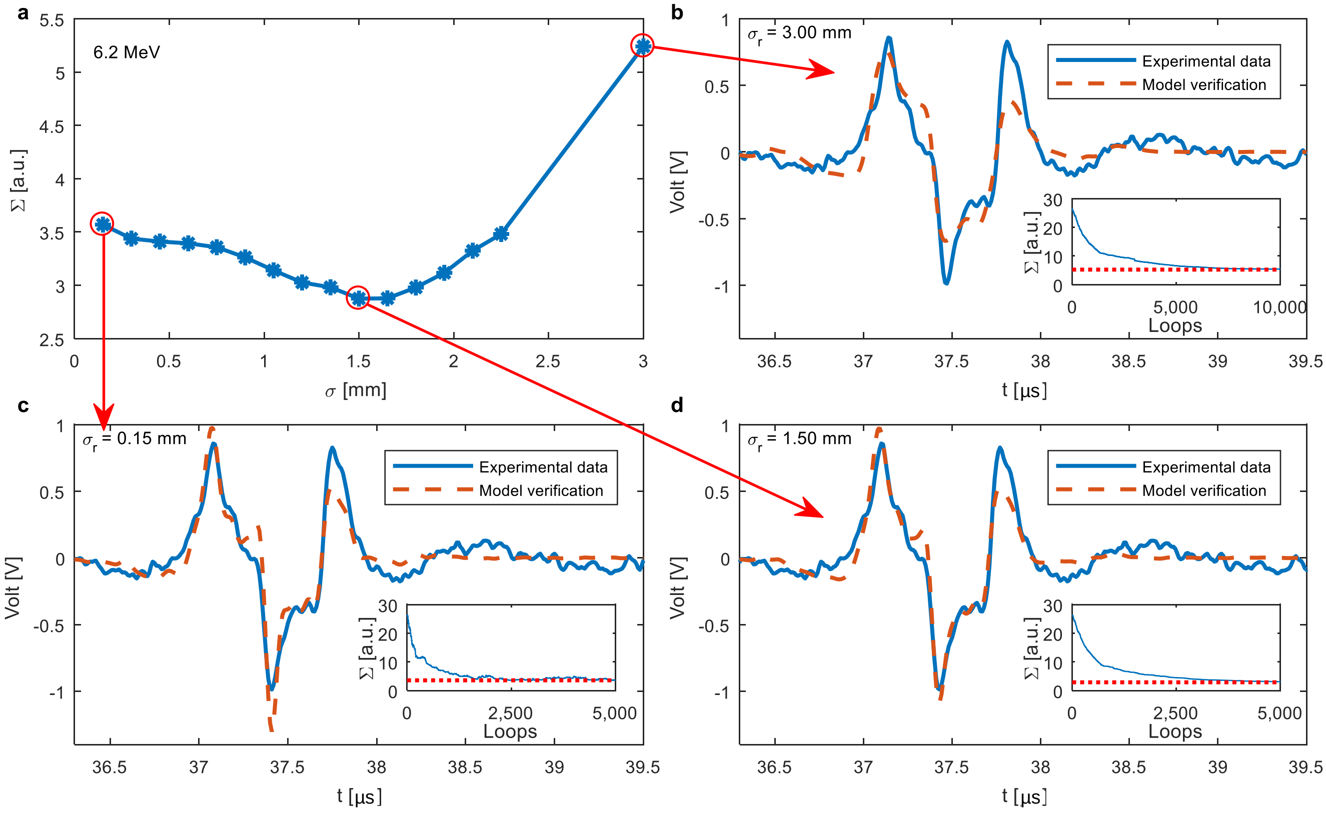

Supplementary figure 5 | Evaluation of different ion bunch diameters for 6.2 MeV. a, shows the residual Σ_i in dependency of the bunch diameter. **b,c, and d,** show the experimentally recorded signal (blue) and the calculated signal using the retrieved (simulated annealing) ion energy distribution. As an inset the development of Σ_i with the number of iterations is shown.

The use of I-BEAT at typical conditions for laser ion acceleration experiments

In this section we investigate the functionality of I-BEAT at different condition typically occurring at laser-ion acceleration experiments. We show the performance of I-BEAT measuring a broad band exponential spectrum that is typically obtained close to target without any manipulation of the ion bunch. We also show the performance of I-BEAT in a multi species spectrum, using quadrupoles as charge state separation.

Functionality of I-BEAT close to target measuring a broad energy distribution

In laser ion acceleration typically broad multispecies energy spectra emerging the plasma target[8,9]. We simulated the performance of I-BEAT positioned close to target without any manipulation of the energy distribution (e.g. magnetic quadrupoles). The proton input spectrum was exemplarily taken from[10]. Note that other ion species are typically emitted with significant lower particle numbers and energy and are thus neglected in this consideration. Without the use of charge state separating fields a differentiation of different ion species is not possible. Assuming and opening aperture for the detector with an radius of 3 mm (seems feasible since it supported by the measured data) covers an area of about A = 30 mm^2 and thus the I-BEAT detector was positioned such that 10^9 protons reach the detector. The particle number was chosen to obtain a good signal to noise ratio (knowing the pressure signal of a single proton and the background noise). The measured spectrum provides more than 10^8 protons per msr (all energies summed up). We thus have to cover a steradian Ω of 10 msr. With $\Omega = \frac{A}{d^2}$ and *d* being the distance to the detector yields d = 50 mm and has thus be positioned close to the target. The given input spectrum was used for a calculation of the expected signal (Supplementary Fig. 6 b). This signal was then evaluated with the I-BEAT algorithm and thus the spectrum was evaluated. In Supplementary Fig. 6a the original spectrum is compared to the ones evaluated with I-BEAT. The required signal to noise ratio thus sets a limit to the distance (to the source), where the I-BEAT detector can be placed. I-BEAT can thus function as the typical RCF stack that is positioned close to target, admittedly without measuring the beam profile but offering an online evaluation.

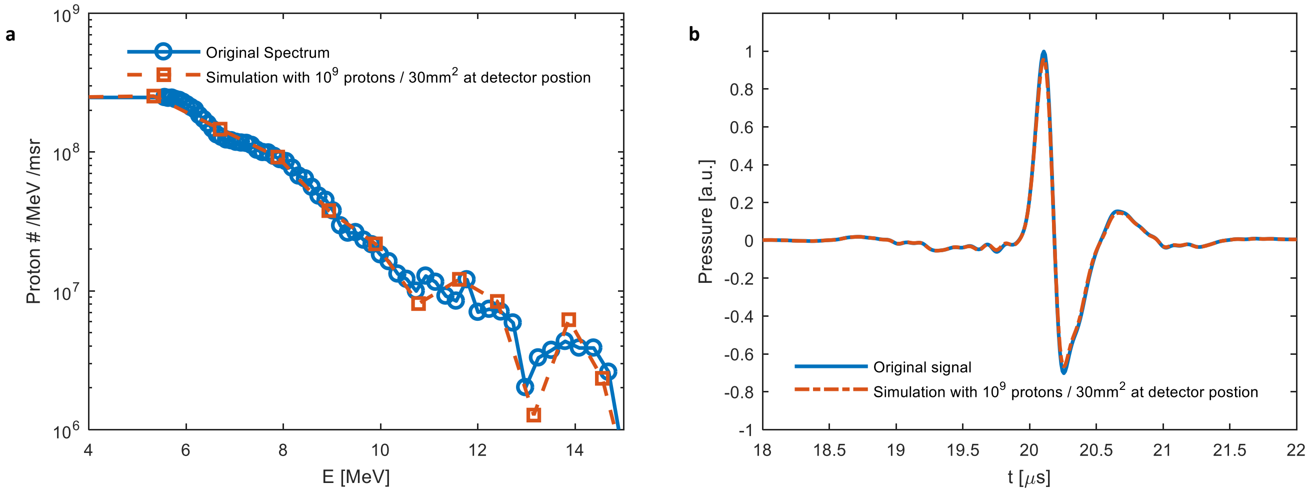

Supplementary figure 6 | I-BEAT positioned close to target. a, Exemplary measured TNSA spectrum taken from[10] (blue curve) and the reconstructed spectrum assuming 10^9 protons at the detector (red curve). **b,** Calculated pressure signal. The blue curve is without any noise disturbance while the red curve is evaluated for 10^9 protons at the detector incorporating the measured noise level. The signal to noise ratio at this high particle number is good and allows the reconstruction of the broad band energy distribution.

Functionality of I-BEAT measuring multiple species

Laser-driven plasmas accelerate not only protons but also other ion species depending on the target material. Especially carbons at different charge states are also emitted from the contamination layer of any target surface. Since the range in the water tank is dependent on the mass (not so much on the initial charge state) and the kinetic energy, I-BEAT is able to assign a certain energy to a certain charge and mass in combination with an energy selective focusing device[11,12] (such as magnetic quadrupoles) and can thus, at least in this configuration also reconstruct the energy distribution of different ion masses and charges in a single shot. An example calculation is presented in the Supplementary Fig. 7. We assume flat spectra of carbon ions with charge states 4, 5 and 6, as well as protons. The QP-doublet focusses all ions with the same synchrotron radius to the same point as depicted in Supplementary Fig. 7a. A calculation of the signal when such a multispecies ion bunch is measured with I-BEAT is performed and the expected acoustic signal is shown in Supplementary Fig. 7b and 7c. One can clearly distinguish the contributions of the different ions to the acoustic wave form and hence measurement of this waveform will allow for reconstructing the complete information. Of course, the information of the complete ion spectra emitted from the target remain inaccessible (as the QPs filter out ions which are too far of the design energy which is focused). I-BEAT will not replace the currently and also really valuable techniques of characterising the composition of the ion-spray emitted from the target, such as provided by Thomson parabola spectrometers[13,14]. But it will be an additional and complementary option to measure ion energies that will give an experimentalist a new, very powerful, tool for future research at the application site of high flux ion bunches.

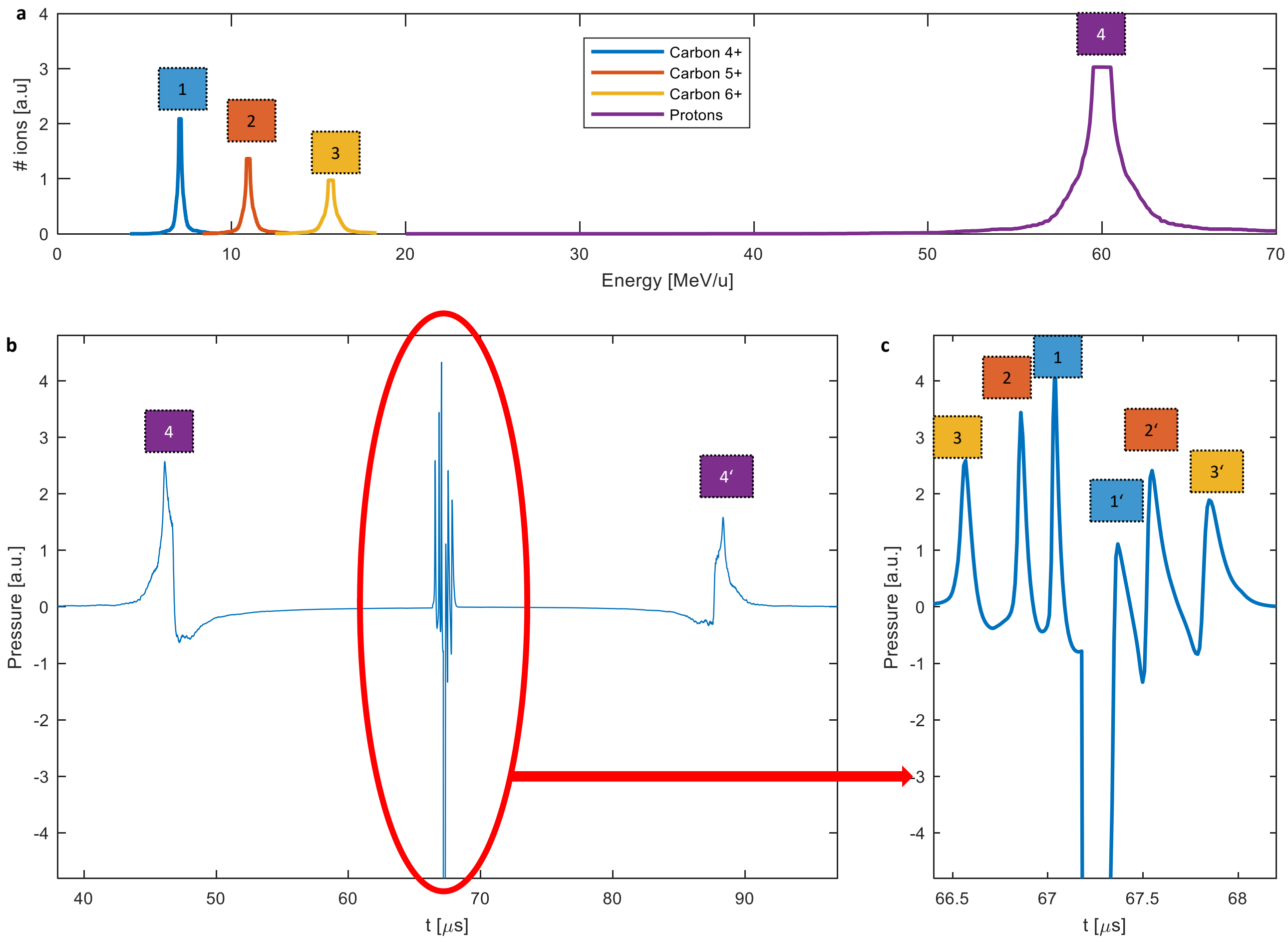

Supplementary figure 7| Multispecies in combination with magnetic quadrupoles. a, Multispecies ion energy distribution selected by quadrupoles, set to a design energy of 60 MeV protons. **b,** Simulated acoustic trace generated by the spectrum in **a**. **c,** Enlargement of the central part of **b** (highlighted with red). The carbon ions do not penetrate far into the water but are still well separated in the oscilloscope trace. Since the peaks are well separated, I-BEAT is capable of reconstructing the complete information.

References for the Supplementary Material

1. Assmann, W. *et al.* The Munich MP tandem. *Nucl. Instrum. Methods* **122,** 191–203 (1974).

2. Rienstra, S. W. & Hirschberg, A. An introduction to acoustics. *Eindh. Univ. Technol.* **18,** 19 (2004).

3. Dowling, A. P. & Ffowcs Williams, J. E. *Sound and sources of sound*. (Ellis Horwood, 1983).

4. Yang, R. Development of an Energy Spectrometer based on Thermoacoustics for Laser Accelerated Ions. (Ludwig-Maximilian-Universität, 2017).

5. Caballero, M. A. A., Rosenthal, A., Buehler, A., Razansky, D. & Ntziachristos, V. Optoacoustic determination of spatio- temporal responses of ultrasound sensors. *IEEE Trans. Ultrason. Ferroelectr. Freq. Control* **60,** 1234–1244 (2013).

6. Ziegler, J. F., Ziegler, M. D. & Biersack, J. P. SRIM - The stopping and range of ions in matter (2010). *Nucl. Instrum. Methods Phys. Res. B* **268,** 1818–1823 (2010).

7. Kirkpatrick, S., Gelatt, C. D. & Vecchi, M. P. Optimization by Simulated Annealing. *Science* **220,** 671–680 (1983).

8. Macchi, A., Borghesi, M. & Passoni, M. Ion acceleration by superintense laser-plasma interaction. *Rev. Mod. Phys.* **85,** 751–793 (2013).

9. Daido, H., Nishiuchi, M. & Pirozhkov, A. S. Review of laser-driven ion sources and their applications. *Rep. Prog. Phys.* **75,** 056401 (2012).

10. Bin, J. H. *et al.* Ion Acceleration Using Relativistic Pulse Shaping in Near-Critical-Density Plasmas. *Phys. Rev. Lett.* **115,** (2015).

11. Rösch, T. F. *et al.* Considerations on employing a PMQ-doublet for narrow and broad proton energy distributions. *Curr. Dir. Biomed. Eng.* **3,** (2017).

12. Busold, S. *et al.* Focusing and transport of high-intensity multi-MeV proton bunches from a compact laser-driven source. *Phys. Rev. Spec. Top. - Accel. Beams* **16,** (2013).

13. Cobble, J. A. *et al.* High-resolution Thomson parabola for ion analysis. *Rev. Sci. Instrum.* **82,** 113504 (2011).

14. Harres, K. *et al.* Development and calibration of a Thomson parabola with microchannel plate for the detection of laser-accelerated MeV ions. *Rev. Sci. Instrum.* **79,** 093306 (2008).
